# Thoracic injection of low-dose interleukin-2 as an adjuvant therapy improves the control of the malignant pleural effusions: a systematic review and meta-analysis base on Chinese patients

**DOI:** 10.1186/s12885-018-4581-5

**Published:** 2018-07-06

**Authors:** Liping Han, Qiufang Jiang, Wei Yao, Tian Fu, Qingdi Zeng

**Affiliations:** 1 Department of respiratory Medicine, Jining NO.1 People’s Hospital, Jining, China; 2 General surgery, Kanzhuang Township Health Center, Zoucheng, China; 3 Department of Clinical Laboratory, Jining NO.1 People’s Hospital, NO.6, Jiankang Road, Jining City, Shandong Province 272011 People’s Republic of China

**Keywords:** Malignant pleural effusions, Interleukin-2, IL-2, Cisplatin, Thoracic injection, Meta-analysis

## Abstract

**Background:**

Interleukin-2 (IL-2) is an important immunotherapy cytokine for various diseases including cancer. Some studies reported the efficacy and safety on cisplatin combined with IL-2 versus cisplatin alone for treating malignant pleural effusion (MPE) through thoracic injection.

**Methods:**

We searched these studies from medical electronic database. A total of 18 studies that met the inclusion criteria were recruited in this meta-analysis. Pooled odds ratios (OR) with 95% confidence intervals (CI) were determined by the fixed effects model of meta-analysis.

**Results:**

The objective response rate (ORR) and disease control rate (DCR) of cisplatin plus IL-2 for controlling MPE was significantly higher than that of cisplatin alone (*p* < 0.001). In addition, compared with cisplatin alone, the presence of IL-2 improved the quality of life (QOL) of patients with MPE (*p* < 0.001). Although the use of IL-2 seemed to increase the probability of fever in patients (*p* = 0.001), it did not lead to extra other side effects (AEs) including myelotoxicity, nausea/vomiting and chest pain (*p* > 0.05).

**Conclusions:**

The low-dose IL-2 improved the ORR, DCR and QOL of patients in the treatment of MPE. Although it may cause fever in patients, it did not increase other AEs.

## Background

Clinically, some malignancies are often associated with malignant pleural effusion (MPE). During tumor progression, tumor cells often invade the pleura, causing destruction of the pleural structure and clogging of the lymphatic vessels, which results in increased pleural microvascular permeability and ultimately pleural effusion [1, 2]. Studies have shown that the incidence of MPE caused by lung cancer is about 7–23, and 15% of cancer deaths are closely related to MPE. MPE often leads to exacerbations of blood-gas exchange in patients with dyspnoea and worsening quality of life (QOL), leading to a decrease in survival. In fact, the treatment of MPE is clinically very difficult [3]. Traditional treatments for MPE include fluid drainage, pleural adhesions, and drug infusion into the chest cavity. Currently, the mainstream view is that thoracic injection of some agents can be used to control MPE, mainly because this mode of administration results in significantly higher intrathoracic drug concentrations than intravenous injection [3, 4]. Today, to find new tactics and effective therapies for controlling MPE remains challenging. However, it is currently trend to explore novel agents that have therapeutic selectivity and are capable of preferentially killing cancer cells without significant toxicity to normal cells [2].

Interleukin-2 (IL-2) is a small 15.5-kDa four α-helical bundle cytokine, which is produced predominately by antigen-simulated cluster of differentiation 4 (CD4+) T cells, while it can also be produced by cluster of differentiation 8 (CD8+) cells, natural killer (NK) cells, and activated dendritic cells [5]. IL-2 is a thymus dependent lymphocyte growth factor, which regulates the proliferation and differentiation of lymphocytes [6]. Especially, it plays a major role in the growth and proliferation of NK and thymus dependent lymphocyte cells, thus it has been introduced to treat various diseases including cancer [7]. Despite some potential side effects, IL-12 has been shown to have more potent effects when used with whole tumor cell vaccines [8]. Generally, it has been thought to play a role in the activation of the immune system, which may be an effective method of eradicating cancer. As a monotherapy, IL-2 has been shown to mediate tumor regression and has been used for treating metastatic renal cell carcinoma and metastatic melanoma [9, 10].

IL-2 alone or in combination with other anti-cancer therapies have brought some survival benefits to advanced cancer patients [5]. A recent meta-analysis seems to support the use of IL-2 in combination with chemotherapy in solid tumors other than melanoma and renal cancer and reports that there is a trend toward better prognosis in the response in several solid tumors, especially for colorectal cancers [11]. Another meta-analysis also shows that IL-2 combination therapy is effcacious in treating non-small cell lung cancer (NSCLC) and improves overall survival and did not show significant toxic reactions [12]. In China, IL-2 has been approved for the treatment of MPE since 1998. These years, some randomized controlled trials (RCTs) have specially explored the clinical efficacy and safety of IL-2 combined with cisplatin versus cisplatin alone in treating MPE through thoracic injection. So we performed a systemic review and meta-analysis to evaluate the efficacy and safety of IL-2 for treating MPE.

## Methods

### Searching of studies

We searched relevant RCTs regarding treating MPE by IL-2 and cisplatin from the databases of Medline/PubMed, EMBASE, Cochrance Library, Web of Science, Chinese Journal Full-text Database. The search period was from the start of each database up to September 2017. The search language was set to English and Chinese. The implementation of the retrieval was carried out by combining keywords and keywords. The search terms are as follows: “malignant pleural effusions”, “MPEs”, “malignant pleural effusion”, “MPE”, “interleukin-2”, “IL-2”, “interleukin-II”, “IL-II”, “cisplatin”, “randomized controlled trial”, “RCTs”, “cancerous pleural effusion”, “chemotherapy”, “thoracic injection”, “pleural injection” and “thoracic injection”. If the retrieved literature suggested some of the key references, we reviewed these documents further. Two of the authors independently searched and screened the literature. If two authors have conflicts on whether to keep one study, all authors discussed it together and made a decision. If necessary, we contact the researcher via e-mail and telephone to confirm the information.

### Inclusion criteria

Inclusion criteria for this systematic review: (1) must be RCTs; (2) must compare cisplatin plus IL-2 with cisplatin alone for controlling the MPE; (3) must have a pathologically defined diagnosis of MPE or pleural metastasis through pleural fluid cytology or pleural tissue biopsies; (4) must be medium to large of MPE; (5) drugs must be given through thoracic injection; (6) the MPE must be drained as far as possible before giving the medication; (7) patients were not given systemic chemotherapy or radiotherapy at the same time or within one month; (8) efficacy evaluation must be determined by WHO criteria or Response Evaluation Criteria In Solid Tumors (RECIST), improvement of QOL (quality of life) must be assessed by Kamofsky scoring criteria (KPS), and adverse reactions (AEs) must be evaluated by WHO Recommendations for Grading of Acute and Subacute Toxicity; (9) good balance between two groups must be displayed, regardless of gender, age, stage, pathological type, were comparable; (10) except for the interventions thoracic injection, the other support treatments in two groups should be the same and (11) the total cases in each study must be greater than or equal to 60.

### Exclusion criteria

The following situations must be excluded: (1) animal models, review articles, meeting reports and other non-first-hand information; (2) patients were treated with antineoplastic agents by intravenous or oral administration during study period; (3) within one month prior to the start of the study, patients received anti-cancer drugs and biological agents via thoracic injection; (4) uncontrolled single arm study; (5) important research indicators have not been better reflected such as complete response (CR), partial response (PR), stable disease (SD), progressive disease (PD), quality of life (QOL), and adverse effects (AEs); (6) the research program was sponsored by the manufacturer or research funding was provided by the manufacturer; (7) duplicate publication and (8) the description of thoracic injection method and drug dose was unclear.

### Research data extraction and analysis

General information were showed in Table 1: (1) authors and reporting time; (2) the number and grouping of patients included in the study, gender and age of the patient, the type of tumor causing MPE, the amount of MPE, and the score of the patient’s physique; (3) intervention grouping and drug management; and (4) dosage and interval. Key research indicators: (1) indicators that reflected clinical efficacy included CR, PR, SD, PD and improvement rate of QOL; (2) objective response rate (ORR) = CR + PR/overall cases; disease control rate (DCR) = CR + PR + SD/overall cases; non-response rate (NRR) = SD + PD/overall cases; (3) AEs.

**Table 1:** Data analysis of included studies

Study	*N*	Male	Female	Age (average)	Resource of tumor						Volume of MPE(N)	Quality of Life (KPS)	End point
					NSCLC	SCLC	Gastrointestinal tumor	Breast cancer	Lymphoma	Others			
Changjie H 2001 [15]	60	44	16	30–76	27		4	11	10	8	Moderate-large	> 60	ORR, DCR, AEs
Xiuzhi Y 2001 [16]	60	24	36	40–75	56		0	0	0	4	–	–	ORR, DCR, AEs
Zhuo S 2004 [17]	62	–	–	–	55		0	0	0	7	–	> 50	ORR, DCR, QOL, AEs
Junyan W 2005 [18]	82	57	25	33–78	68		0	10	0	4	–	> 50	ORR, DCR, AEs
Haiying X 2009 [19]	63	35	28	35–71	63		0	0	0	0	–	> 60	ORR, DCR, AEs
Xiaoxia H 2009 [20]	72	–	–	–	65		0	0	0	7	Small (8) Moderate (39) Large (25)	> 60	ORR, DCR, QOL, AEs
Lizheng C 2009 [21]	86	62	24	25–75	23	0	27	17	8	11	Large(29) Moderate(57)	> 60	ORR, DCR, AEs
Jinguang C 2009 [22]	62	28	34	41–77	52	10	0	0	0	0	–	> 60	ORR, DCR, AEs
Junfeng W 2010 [23]	82	48	34	30–80	54		0	15	0	13	Large(56) Moderate(26)	> 60	ORR, DCR, AEs
Jingping Z 2010 [24]	124	80	44	47–73	124		0	0	0	0	Large(84) Moderate(40)	> 60	ORR, DCR, AEs
Cheng X 2010 [25]	62	28	34	19–81	40		0	9	13		–	> 70	ORR, DCR, AEs
Fang S 2011 [26]	60	31	29	38–76	41	8	2	7	2	0	Large(34) Moderate(17)	> 40	ORR, DCR, QOL, AEs
Xueling L 2011 [27]	68	40	28	30–75	40		10	8	0	10	–	> 60	ORR, DCR, AEs
Yan Q 2011 [28]	76	45	31	46–77	76		0	0	0	0	–	> 60	ORR, DCR, AEs
Li J 2013 [29]	73	40	33	35–74	31		16	26	0	0	Large(41) Moderate(32)	> 60	ORR, DCR, QOL, AEs
Lijie H 2014 [30]	60	38	22	39–78	60		0	0	0	0	Large	> 50	ORR, DCR, QOL, AEs
Miao H 2016 [31]	61	40	21	38–75	42		0	8	6	5	Large	> 60	ORR, DCR, AEs
Baohua Y 2017 [32]	66	35	31	42–76	–	–	–	–	–	–	–	> 60	RR, DCR

*N* number of patients, *NSCLC* non-small cell lung cancer, *SCLC* small cell lung cancer, *MPE* malignant pleural effusions, *KPS* karnofsky physical status score, *ORR* objective response rate, DCR, disease control rate, *QOL* quality of life, *AEs* adverse effects

### Efficacy evaluation criteria for treating MPE used in the included studies

All studies have adopted the same criteria recommended by WHO for evaluating the treatment efficacy of MPE [13]. CR: pleural effusion completely disappeared, and at least 4 weeks or more; PR: pleural effusion was significantly reduced (> 50%) and maintained for more than 4 weeks; SD: reduced pleural effusion > 50% or increased < 25%; PD: pleural effusion increased by > 25%.

### The implementation process of intervention

Study design: (1) RCTs of cisplatin combined with IL-2 versus cisplatin alone through intrapleural injection for treating MPE; (2) observation group = thoracic injection of IL-2 and cisplatin, control group = cisplatin alone. Drug management: (1) the dosage of IL-2 was 1 to 3 million units per time in different studies, which were showed in Table 2 in detail; (2) frequency of thoracic injection was 1/week, and at least 2 cycles or pleural effusion disappeared, which were showed in Table 2 in detail; and (3) patient’s withdrawal and loss of follow-up during the study have been reported.

**Table 2:** Assessment method of administration of included studies

Study	Trial group (*N*)	Control Group(*N*)	Interventions (Groups)		Treatment cycle	Termination of treatment
			Cisplatin+IL-2	Cisplatin alone		
Changjie H 2001 [15]	30	30	Cisplatin: 50 mg + NS 50 mL IL-2: 2 million units+NS 30 mL	Cisplatin 50 mg + NS 50 mL	1/week	> 2 cycles, or pleural effusion disappeared
Xiuzhi Y 2001 [16]	40	20	Cisplatin: 40 mg + NS 50 mL IL-2: 2 million units+NS 30 mL	Cisplatin 40 mg + NS 50 mL	1/week	> 2 cycles, or pleural effusion disappeared
Zhuo S 2004 [17]	32	30	Cisplatin: 60 mg + NS 30 mL IL-2: 2 million units+NS 30 mL	Cisplatin 60 mg + NS 30 mL	1/week	> 2 cycles, or pleural effusion disappeared
Junyan W 2005 [18]	48	34	Cisplatin: 80-100 mg + NS 30 mL IL-2: 2–3 million units+NS 30 mL	Cisplatin 80-100 mg + NS 30 mL	1/week	> 3 cycles, or pleural effusion disappeared
Haiying X 2009 [19]	35	28	Cisplatin: 60 mg + NS 40 mL IL-2: 2 million units+NS 20 mL	Cisplatin 60 mg + NS 40 mL	1/week	> 2 cycles, or pleural effusion disappeared
Xiaoxia H 2009 [20]	37	35	Cisplatin: 100 mg + NS 50 mL IL-2: 1 million units+NS 30 mL	Cisplatin 100 mg + NS 50 mL	1/week	> 2 cycles, or pleural effusion disappeared
Lizheng C 2009 [21]	46	40	Cisplatin: 50 mg + NS 50 mL IL-2: 2–3 million units+NS 50 mL	Cisplatin 50 mg + NS 50 mL	1/week	> 2 cycles, or pleural effusion disappeared
Jinguang C 2009 [22]	31	31	Cisplatin: 60 mg + NS 50 mL IL-2: 2 million units+NS 50 mL	Cisplatin 60 mg + NS 50 mL	1/week	> 3 cycles, or pleural effusion disappeared
Junfeng W 2010 [23]	41	41	Cisplatin: 60-80 mg + NS 50 mL IL-2: 2–3 million units+NS 50 mL	Cisplatin 60-80 mg + NS 50 mL	1/week	> 3 cycles, or pleural effusion disappeared
Jingping Z 2010 [24]	63	61	Cisplatin: 40-60 mg + NS 50 mL IL-2: 1 million units+NS 40 mL	Cisplatin 40–60 + NS 40 mL	1/week	> 3 cycles, or pleural effusion disappeared
Cheng X 2010 [25]	31	31	Cisplatin: 60-100 mg + NS 50 mL IL-2: 1–2 million units+NS 50 mL	Cisplatin 60-100 mg + NS 50 mL	1/week	> 1 cycles, or pleural effusion disappeared
Fang S 2011 [26]	30	30	Cisplatin: 60 mg + NS 40 mL IL-2: 2 million units+NS 40 mL	Cisplatin 60 mg + NS 40 mL	1/week	> 3 cycles, or pleural effusion disappeared
Xueling L 2011 [27]	34	34	Cisplatin: 60 mg + NS 50 mL IL-2: 2 million units+NS 50 mL	Cisplatin 60 mg + NS 50 mL	1/week	> 3 cycles, or pleural effusion disappeared
Yan Q 2011 [28]	41	35	Cisplatin: 60 mg + NS 40 mL IL-2: 2 million units+NS 40 mL	Cisplatin 60 mg + NS 40 mL	1/week	> 3 cycles, or pleural effusion disappeared
Li J 2013 [29]	38	35	Cisplatin: 60-80 mg + NS 50 mL IL-2: 1–2 million units+NS 20 mL	Cisplatin 60-80 mg + NS 50 mL	1/week	> 3 cycles, or pleural effusion disappeared
Lijie H 2014 [30]	30	30	Cisplatin: 40-60 mg + NS 50 mL IL-2: 2 million units+NS 50 mL	Cisplatin 40-60 mg + NS 50 mL	1/week	> 2 cycles, or pleural effusion disappeared
Miao H 2016 [31]	31	30	Cisplatin: 60 mg + NS 50 mL IL-2: 2–3 million units+NS 30 mL	Cisplatin 60 mg + NS 50 mL	1/week	> 3 cycles, or pleural effusion disappeared
Baohua Y 2017 [32]	33	33	Cisplatin: 70-80 mg + NS 50 mL IL-2: 2–3 million units+NS 30 mL	Cisplatin 70-80 mg + NS 50 mL	1/week	> 3 cycles, or pleural effusion disappeared

*N* numbers of patients, *IL-2* interleukin-2, *NS* normal saline

### The overall quality evaluation of included studies

The quality evaluation of literature are performed based on the Cochrane Handbook of Systematic Review [14]. Indicators: (1) the generation of random sequences; (2) the hiding of distribution programs; (3) blindness (for patient and outcome evaluator); (4) data integrity; (5) selective reporting of results; (6) other sources of bias. Two reviewers independently evaluated the literature. Disagreements arising during the evaluation process were resolved through discussion and negotiation with another reviewer’s participation.

### Statistical methods and analysis

The statistics involved in this study are as follows: (1) we identified the heterogeneity of studies using two statistical methods, Chi-square test and I^2^-statistic test. When the *P* value for the chi-square test was greater than 0.10 and the I^2^ value was less than or equal to 50%, indicating that a heterogeneity did not appear in these studies, we chose the fixed effect method of meta-analysis. Otherwise, we explained the causes of heterogeneity and took a random effect model to complete the meta-analysis; (2) we used the odds ratio and the 95% confidence interval (CI) to estimate the statistical effect of meta-analysis on the dichotomous variables; (3) We determined the overall effect using Z-scores, with significance being set at *p* < 0.05; (4) we deleted one study every time to re-estimate the overall effect; if the results did not change, indicating the conclusion was more stable and credible; (5) we employed funnel plot analysis, Egger’s test and Begg’s test to decide the possibility of publication bias; (6) the statistics of continuous variables were analyzed by SPSS software (version 20.0, IBM Corporation); (7) two software, Revman 5.2 (the cochrane collaboration) and Stata version 14.0 (Stata Corporation, TX, USA) were used to perform meta-analysis. When the *p* value was less than 0.05, the difference was considered statistically significant.

## Results

### A total of 18 studies were recruited in this meta-analysis

**Fig. 1:**
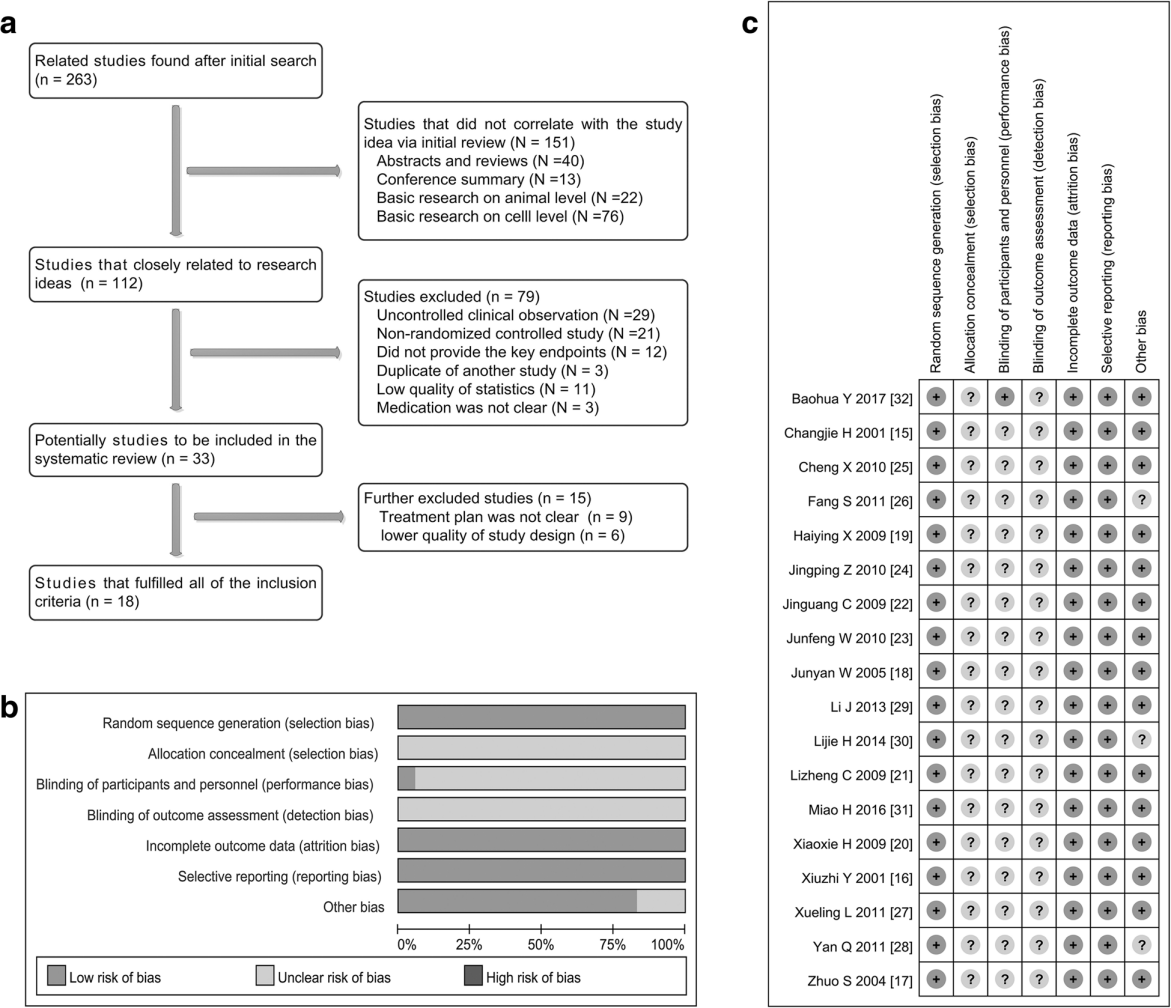
Screening and identification of included studies. **a** A total of 18 articles that met the inclusion criteria were included in meta-analysis, which were searched from the database of Medline/PubMed, EMBASE, Cochrance Library, Web of Science, Chinese Journal Full-text Database and Chinese Sci-Tech Journals Database. **b**, **c** Through a comprehensive analysis according to the Cochrane Handbook of Systematic Review, we found that except for a study that was low risk, the other studies did not have obvious biases

At first, a total of 263 documents were initially retrieved. Subsequently, 112 of them that might meet the criteria were screened out (excluded documents are mainly abstract, conference summary and basic research). After reading the full article, 79 articles were excluded once again, including: non-control clinical observation (29), non-randomized controlled study (21), unclear outcome measures (12), repeated published articles (3), low-level statistical analysis (11) and not clear description on administration of the drug (3). Thus, 33 studies potentially met our inclusion criteria. Unfortunately, we had to discard 15 of them because of the following problems: unreasonable treatment plan (9) and low quality of study design (6). In the end, a total of 18 studies [15–32] met our inclusion criteria exactly and were included in the analysis (Fig. 1a).

### General characteristics of included studies reflected the balance of various parameters between the two groups

As shown in Table 1, a total of 18 [15–32] studies included 1279 patients. Male patients accounted for 52.8% and women accounted for 47.2%. The youngest patient was 19 years old [25] and the oldest was 81 years old [25]. Although some studies do not provide detailed information on the cause of MPE, the main causes included: lung cancer (73.1%), gastrointestinal malignancies (4.6%), breast cancer (8.7%), lymphomas (3%) and other malignant tumor (10.6%). Most of studies reported that the patient’s pleural effusion volume was medium to large and all studies [15–32] reported the data on patients’ physical status scores (at least KPS > 40). Some studies provided data on QOL [17, 20, 26, 29, 30], but all studies [15–32] reported significant data on the efficacy evaluation, which included RR, DCR and AEs.

### Included studies showed a good consistency and comparability in administration and implementation of study

As shown in Table 2, this meta-analysis consisted of 1279 patients, 671 patients in the treatment group and 608 patients in the control group. Drugs used the treatment group were cisplatin plus IL-2 and the control group was cisplatin alone. Thoracic injection was the only route of administration. Cisplatin doses ranged from 40 to 80 mg each time and IL-2 was 1 to 3 million units each time in different studies, which were showed in Table 2 in detail. Both drugs were dissolved in physiological saline (natural saline) for use, and the dose of physiological saline was 30-40 mL. All studies were administered once a week for at least 2 cycles or the pleural effusion disappeared, which were showed in Table 2 in detail.

### Quality evaluation showed that included studies had a moderate to high study quality

Two authors of us independently assessed and determined the design quality of each research. As shown in Table 3, all studies [15–32] included in this meta-analysis described the way that random sequences were generated. The blinding description of one study was clear [32], the rest of the studies did not describe the allocation hiding and the blinding implementation. All studies [15–32] had complete outcome data and no selective reported results. Through a comprehensive analysis, we found that one study [32] had very low risk, and the other studies did not show certain bias risk (Fig. 1b, c and f).

**Table 3:** Design quality of included trials

Study	Region	Sequence generation	Allocation concealment	Blind	Outcome data	Selective outcome reporting	Other sources of bias	ITT	Risk of bias
Changjie H 2001 [15]	Single center	Random number table	Unclear	Unclear	Yes	No	Unclear	Yes	Unclear risk of bias
Xiuzhi Y 2001 [16]	Single center	Random number table	Unclear	Unclear	Yes	No	Unclear	Yes	Unclear risk of bias
Zhuo S 2004 [17]	Single center	Random number table	Unclear	Unclear	Yes	No	Unclear	Yes	Unclear risk of bias
Junyan W 2005 [18]	Single center	Random number table	Unclear	Unclear	Yes	No	Unclear	Yes	Unclear risk of bias
Haiying X 2009 [19]	Single center	Random number table	Unclear	Unclear	Yes	No	Unclear	Yes	Unclear risk of bias
Xiaoxia H 2009 [20]	Single center	Random number table	Unclear	Unclear	Yes	No	Unclear	Yes	Unclear risk of bias
Lizheng C 2009 [21]	Single center	Random number table	Unclear	Unclear	Yes	No	Unclear	Yes	Unclear risk of bias
Jinguang C 2009 [22]	Single center	Random number table	Unclear	Unclear	Yes	No	Unclear	Yes	Unclear risk of bias
Junfeng W 2010 [23]	Single center	Random number table	Unclear	Unclear	Yes	No	Unclear	Yes	Unclear risk of bias
Jingping Z 2010 [24]	Single center	Random number table	Unclear	Unclear	Yes	No	Unclear	Yes	Unclear risk of bias
Cheng X 2010 [25]	Single center	Random number table	Unclear	Unclear	Yes	No	Unclear	Yes	Unclear risk of bias
Fang S 2011 [26]	Single center	Random number table	Unclear	Unclear	Yes	No	Unclear	Yes	Unclear risk of bias
Xueling L 2011 [27]	Single center	Random number table	Unclear	Unclear	Yes	No	Unclear	Yes	Unclear risk of bias
Yan Q 2011 [28]	Single center	Random number table	Unclear	Unclear	Yes	No	Unclear	Yes	Unclear risk of bias
Li J 2013 [29]	Single center	Random number table	Unclear	Unclear	Yes	No	Unclear	Yes	Unclear risk of bias
Lijie H 2014 [30]	Single center	Random number table	Unclear	Unclear	Yes	No	Unclear	Yes	Unclear risk of bias
Miao H 2016 [31]	Single center	Random number table	Unclear	Unclear	Yes	No	Unclear	Yes	Unclear risk of bias
Baohua Y 2017 [32]	Single center	Random number table	Unclear	Clear	Yes	No	Clear	Yes	Low risk of bias

*ITT* intention-to-treat

### Included studies did not show a significant heterogeneity

To compare the short-term efficacy of the two different treatment regimens, the statistical results suggested a chi-square statistic of 8.10 (degrees of freedom = 17; *p* = 0.964) and the I-square statistic (due to heterogeneity in OR changes) value of 0.0%. To compare the AEs of the two different treatment regimens, the results showed a chi-square statistic of 13.68 (degrees of freedom = 24; *p* = 0.954) and the I-square statistic (due to heterogeneity in OR changes) value of 0.0%. These results suggested that there was no significant heterogeneity among these studies. From a clinical design perspective, these studies also had very good homogeneity, and the design and implementation of these studies were well comparable. So we used the fixed effects model of meta-analysis to finish the following analysis.

### Thoracic injection of cisplatin plus IL-2 showed a higher ORR as compared with cisplatin alone

**Fig. 2:**
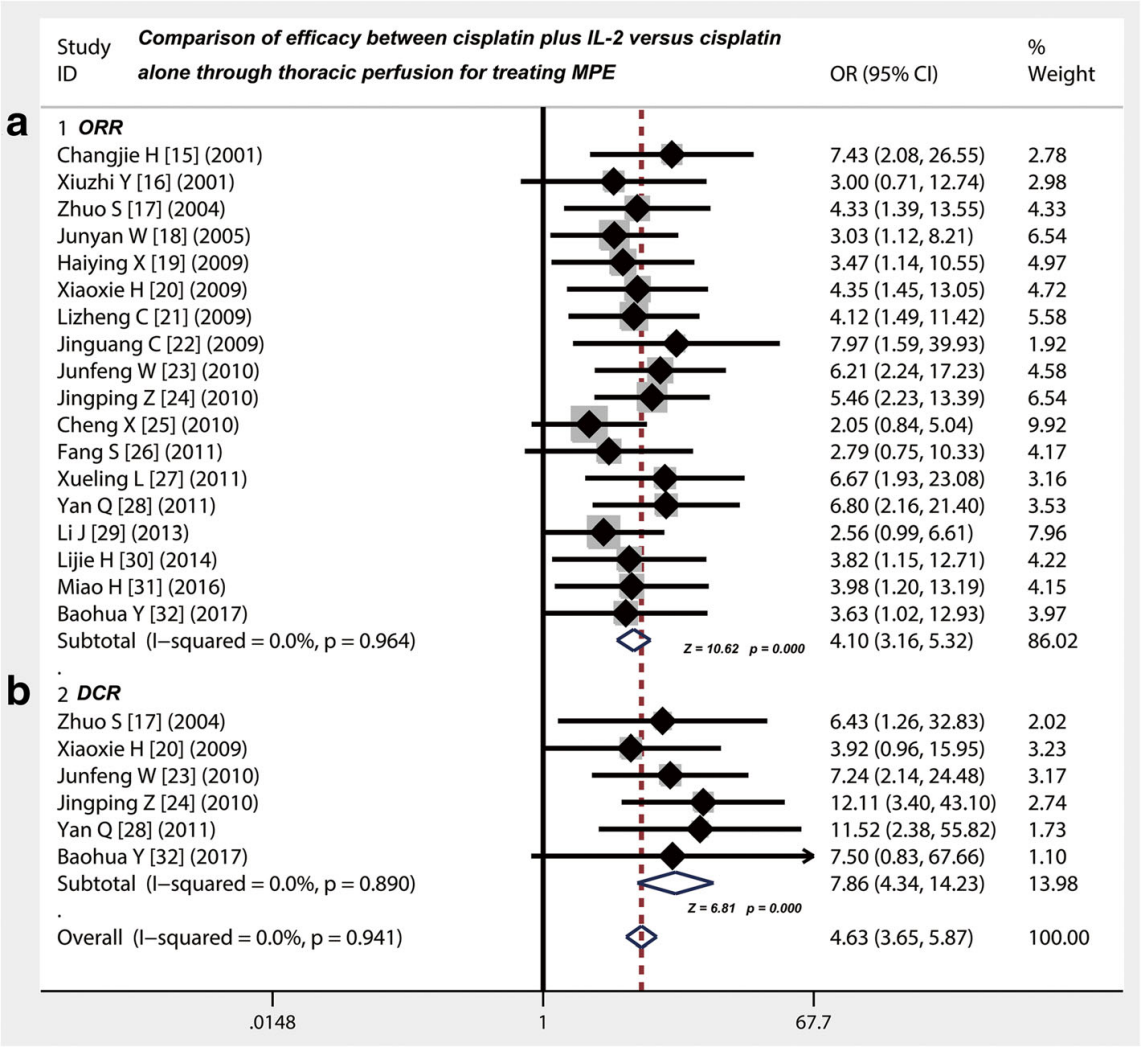
Efficacy comparison of cisplatin combined with IL-2 versus cisplatin alone by thoracic injection for controlling MPE. **a** Thoracic injection of cisplatin combined with IL-2 had a higher ORR compared with cisplatin alone (*p* < 0.001). **b** Thoracic injection of cisplatin combined with IL-2 had a higher DCR compared with cisplatin alone (*p* < 0.001); ORR, objective response rate; DCR, disease control rate; OR, odds ratio

Eighteen studies [15–32] provided data on comparison of ORR between cisplatin plus IL-2 versus cisplatin alone through thoracic injection for treating MPE (Table 4). A meta-analysis of fixed-effects model suggested that the odds ratio (OR) of both was 4.10 (95% CI 3.16 to 5.32; Z value = 10.62, *p* = 0.000), which indicating that the ORR of cisplatin plus IL-2 was higher than that of cisplatin alone (Fig. 2a), responding an absolute 4.1-fold increase.

**Table 4:** Efficacy of IL-2 in treating MPE

Study	Study size (*N*)		Study design		Efficacy of therapy								Improvement of QOL (*N*; %)			
			Group 1	Group 2	Group 1				Group 2				Group 1		Group 2	
	Group 1	Group 2			CR	PR	SD	PD	CR	PR	SD	PD	*N*	%	*N*	%
Changjie H 2001 [15]	30	30	Cisplatin+IL-2	Cisplatin	16	10	4		6	8	16		–	–	–	–
Xiuzhi Y 2001 [16]	40	20	Cisplatin+IL-2	Cisplatin	17	19	5		8	7	5		–	–	–	–
Zhuo S 2004 [17]	32	30	Cisplatin+IL-2	Cisplatin	14	12	4	2	5	10	6	9	20	63	11	37
Junyan W 2005 [18]	48	34	Cisplatin+IL-2	Cisplatin	25	14	9		12	8	14		–	–	–	–
Haiying X 2009 [19]	35	28	Cisplatin+IL-2	Cisplatin	10	18	7		3	12	13		–	–	–	–
Xiaoxia H 2009 [20]	37	35	Cisplatin+IL-2	Cisplatin	15	16	3	3	8	11	7	9	29	78.3	19	54.3
Lizheng C 2009 [21]	46	40	Cisplatin+IL-2	Cisplatin	26	13	7		13	10	17		–	–	–	–
Jinguang C 2009 [22]	31	31	Cisplatin+IL-2	Cisplatin	12	17	2		7	13	11		–	–	–	
Junfeng W 2010 [23]	41	41	Cisplatin+IL-2	Cisplatin	29	4	3	5	7	15	3	16	–	–	–	–
Jingping Z 2010 [24]	63	61	Cisplatin+IL-2	Cisplatin	25	30	5	3	12	22	4	23	–	–	–	–
Cheng X 2010 [25]	41	41	Cisplatin+IL-2	Cisplatin	21	17	3		13	8	20		–	–	–	–
Fang S 2011 [26]	30	30	Cisplatin+IL-2	Cisplatin	9	17	4		7	14	9		26	86.7	24	80
Xueling L 2011 [27]	34	34	Cisplatin+IL-2	Cisplatin	19	11	4		8	10	16		–	–	–	–
Yan Q 2011 [28]	41	35	Cisplatin+IL-2	Cisplatin	12	24	3	2	6	12	4	13	–	–	–	–
Li J 2013 [29]	38	35	Cisplatin+IL-2	Cisplatin	12	13	13		4	11	20		27	71.1	17	48.6
Lijie H 2014 [30]	30	30	Cisplatin+IL-2	Cisplatin	12	13	5		7	10	13		26	86.7	19	63.3
Miao H 2016 [31]	31	30	Cisplatin+IL-2	Cisplatin	11	15	5		7	10	13		–	–	–	–
Baohua Y 2017 [32]	33	33	Cisplatin+IL-2	Cisplatin	11	18	3	1	8	14	6	5	–	–	–	–

N, cases, *IL-2* interleukin-2; Group 1 = IL-2 combined+cisplatin; Group 2 = cisplatin alone, *CR* complete response, *PR* partial response, *SD* stable disease, *PD* progressive disease, *QOL* quality of life

### Thoracic injection of cisplatin plus IL-2 displayed a higher DCR as compared with cisplatin alone

Six studies [17, 20, 23, 24, 28, 32] provided data on comparison of DCR between cisplatin plus IL-2 versus cisplatin alone through thoracic injection for treating MPE (Table 4). The results of meta-analysis showed that the OR of both was 7.86 (95% CI 4.34 to 14.23; Z value = 6.81, *p* = 0.000), which suggesting that cisplatin plus IL-2 increased the DCR for controlling MPE compared with cisplatin alone (Fig. 2b), responding an absolute 7.86-fold increase.

### NRR of cisplatin alone was higher than that of cisplatin plus IL-2 through thoracic injection for treating MPE

**Fig. 3:**
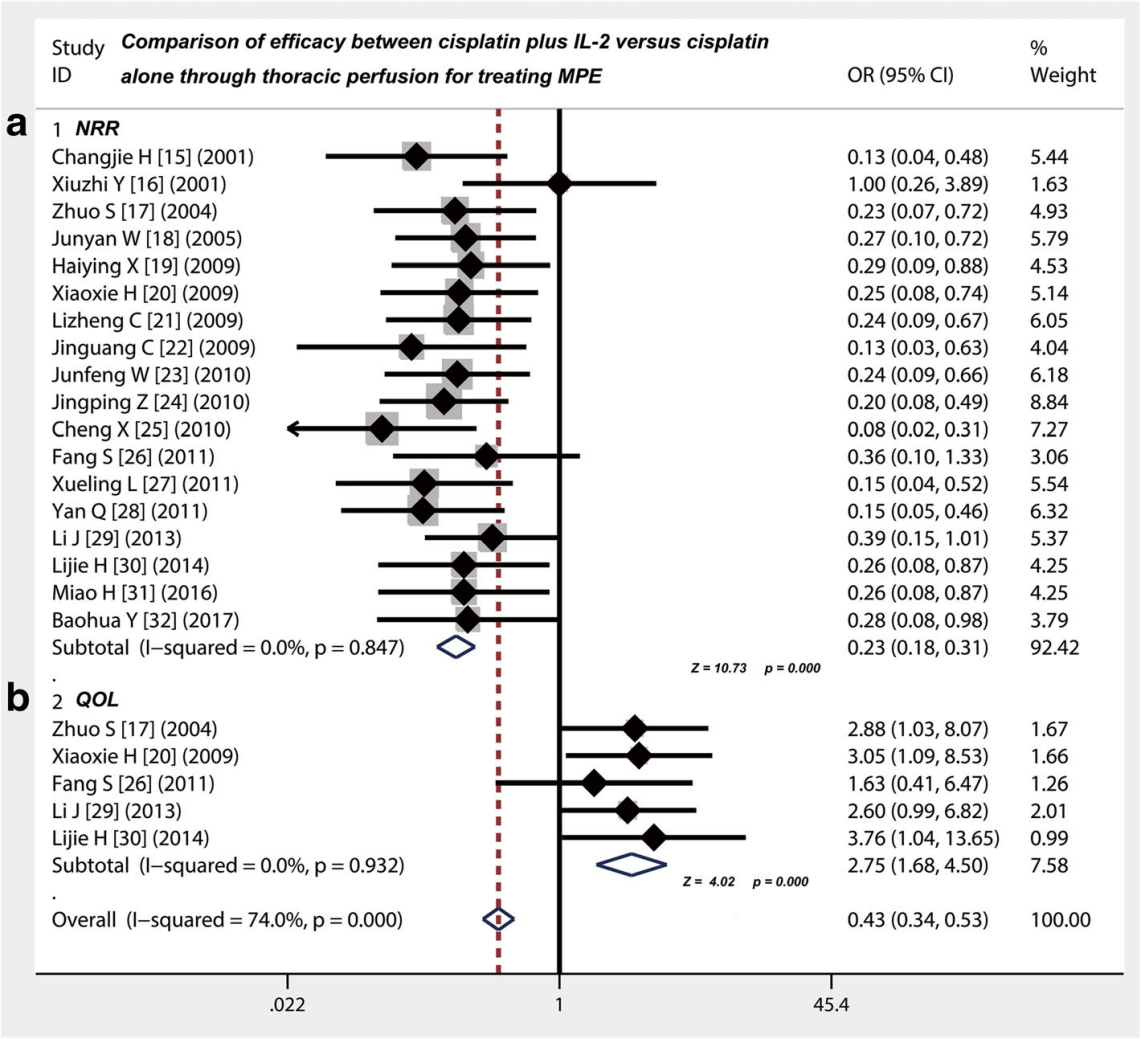
Efficacy comparison of cisplatin combined with IL-2 versus cisplatin alone by thoracic injection for controlling MPE. **a** Thoracic injection of cisplatin alone had a higher NRR compared with cisplatin combined with IL-2 (*p* < 0.001). **b** Thoracic injection of cisplatin combined with IL-2 had a higher QOL compared with cisplatin alone (*p* < 0.001); NRR, non-response rate; QOL, quality of life; OR, odds ratio

Eighteen studies [15–32] provided data on comparison of NRR between cisplatin plus IL-2 versus cisplatin alone through thoracic injection for treating MPE (Table 4). The results of meta-analysis showed that the OR of both was 0.23 (95% CI 0.18 to 0.31; Z value = 10.73, *p* = 0.000), which suggesting that NRR of cisplatin alone was higher than that of cisplatin plus IL-2 through thoracic injection for treating MPE (Fig. 3a), implying that there was a better benefit of cisplatin plus IL-2 than IL-2 alone.

### Thoracic injection of cisplatin plus IL-2 improved the QOL of patients with MPE compared with cisplatin alone

Five studies [17, 20, 26, 29, 30] provided data on comparison of QOL between cisplatin plus IL-2 versus cisplatin alone through thoracic injection for treating MPE (Table 4). The results of meta-analysis showed that the OR of both was 2.75 (95% CI 1.68 to 4.50; Z value = 4.02, *p* = 0.000), which suggested that cisplatin plus IL-2 improved the QOL of patients with MPE compared with cisplatin alone (Fig. 3b), responding an absolute 2.75-fold increase.

### Cisplatin plus IL-2 displayed the same incidence rate on myelotoxicity, nausea/vomiting and chest pain compared with cisplatin alone

**Fig. 4:**
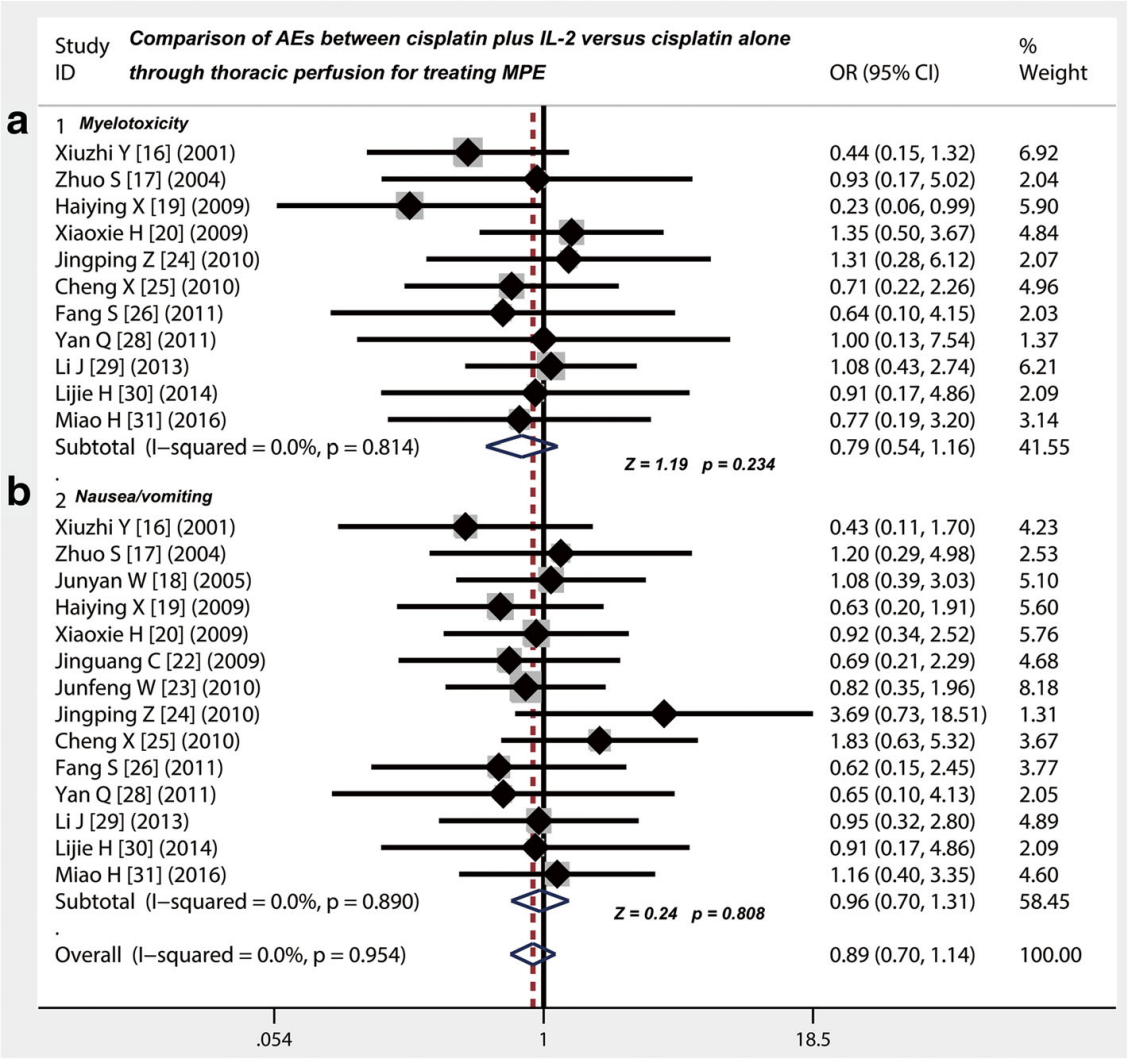
Safety evaluation of cisplatin combined with IL-2 versus cisplatin alone by thoracic injection for controlling MPE. **a** The therapy of cisplatin combined with IL-2 displayed the same incidence rate of myelotoxicity compared with cisplatin alone (*p* > 0.05). **b** The therapy of cisplatin combined with IL-2 had the same incidence of nausea/vomiting compared with cisplatin alone (*p* > 0.05); OR, odds ratio

**Fig. 5:**
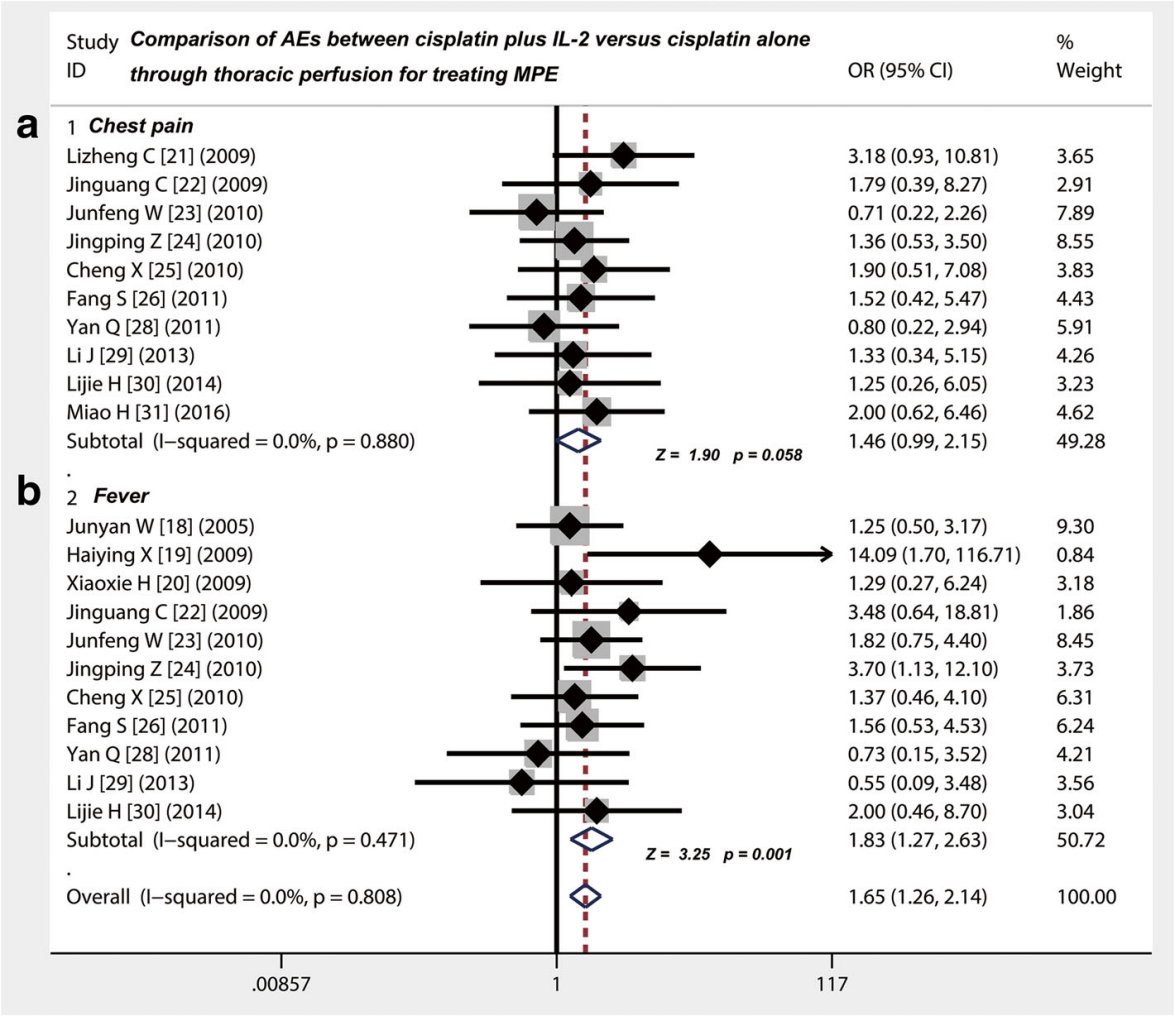
Safety evaluation of cisplatin combined with IL-2 versus cisplatin alone by thoracic injection for controlling MPE. **a** The incidence rate of chest pain in group of cisplatin combined with IL-2 was no difference with cisplatin alone (*p* > 0.05). **b** The incidence rate of the fever in group of cisplatin combined with IL-2 was higher than that in group of cisplatin alone (*p* = 0.001)

Eleven [16, 17, 19, 20, 24–26, 28–31] of the included studies gave a comparison of the incidence of myelotoxicity and nausea/vomiting between cisplatin plus IL-2 versus cisplatin alone through thoracic injection for treating MPE (Table 5). Whether it is myelotoxicity (OR = 0.79; 95% CI 0.54 to 1.16; Z value = 1.19, *p* = 0.234) (Fig. 4a), nausea/vomiting (OR = 0.96; 95% CI 0.7 to 1.31; Z value = 0.24, *p* = 0.808) (Fig. 4b) or chest pain (OR = 1.46; 95% CI 0.99 to 2.15; Z value = 1.90, *p* = 0.058) (Fig. 5a), the two different projects, cisplatin plus IL-2 and cisplatin alone, all displayed the same incidence rate on these AEs.

**Table 5:** Comparison of adverse events between cisplatin combined with IL-2 versus cisplatin alone

Study	Study size (*N*)		Myelotoxicity				Nausea/vomiting				Chest pain				Fever			
			Group 1		Group 2		Group 1		Group 2		Group 1	Group 2			Group 1		Group 2	
	Group 1	Group 2	*N*	%	*N*	%	*N*	%	*N*	%	*N*	%	*N*	%	*N*	%	*N*	%
Changjie H 2001 [15]	30	30	–	–	–	–	13	43.3	0	0	–	–	–	–	10	33.3	0	0
Xiuzhi Y 2001 [16]	40	20	14	35	11	55	5	12.5	5	25	–	–	–	–	–	–	–	–
Zhuo S 2004 [17]	32	30	3	9.3	3	10	5	15.6	4	13.3	–	–	–	–	–	–	–	–
Junyan W 2005 [18]	48	34	–	–	–	–	12	25	8	23.5	–	–	–	–	18	37.5	11	32.4
Haiying X 2009 [19]	35	28	3	9	8	28.6	8	22.9	9	32.1	–	–	–	–	12	36.4	1	4
Xiaoxia H 2009 [20]	37	35	13	35.1	10	25	11	29.7	11	31.4	–	–	–	–	4	10.8	3	8.6
Lizheng C 2009 [21]	46	40	–	–	–	–	–	–	–	–	12	26.1	4	10	–	–	–	–
Jinguang C 2009 [22]	31	31	–	–	–	–	6	20	8	26	5	16	3	10	6	19	2	6
Junfeng W 2010 [23]	41	41	–	–	–	–	19	46.4	21	51.2	6	14.6	8	19.5	21	51.2	15	36.3
Jingping Z 2010 [24]	63	61	4	6.3	3	4.9	7	11.1	2	3.3	12	19.5	9	14.8	13	20.6	4	6.6
Cheng X 2010 [25]	41	41	6	19.4	8	25.8	11	35.5	7	26.6	7	22.6	4	12.9	9	29	7	26.6
Fang S 2011 [26]	30	30	2	6.6	3	10	4	13.3	6	20	7	23.3	5	16.6	12	40	9	30
Yan Q 2011 [28]	34	34	2	4.8	2	5.7	2	4.8	3	8.6	5	12.2	6	17.1	3	7.3	4	11.4
Li J 2013 [29]	41	35	16	42.1	13	37.1	9	23.7	8	22.8	6	15.8	4	11.4	2	7.1	3	8.5
Lijie H 2014 [30]	38	35	3	10	3	10	3	10	3	10	4	13.3	3	10	6	20	3	10
Miao H 2016 [31]	30	30	4	12.9	5	16.6	11	35.5	10	33.3	24	77.4	20	66.6	–	–	–	–
	*P* > 0.05						*P* > 0.05			*P* > 0.05				*P* < 0.05				

*IL-2* interleukin-2, N, cases; Values are given as number of patients (%). Group 1 = cisplatin+IL-2; Group 2 = cisplatin alone

### Thoracic injection of cisplatin plus IL-2 led to a higher possibility of fever than cisplatin alone

Eleven of the included studies showed the data on comparison of the incidence of fever between cisplatin plus IL-2 versus cisplatin alone through thoracic injection for treating MPE (Table 5). We found that compared with cisplatin alone, thoracic injection of cisplatin plus IL-2 led to a higher possibility of fever (OR = 1.83; 95% CI 1.27 to 2.63; Z value = 3.25, *p* = 0.001) (Fig. 5b).

### Sensitivity analysis showed that the presence or absence of any study did not affect the overall statistical performance

**Fig. 6:**
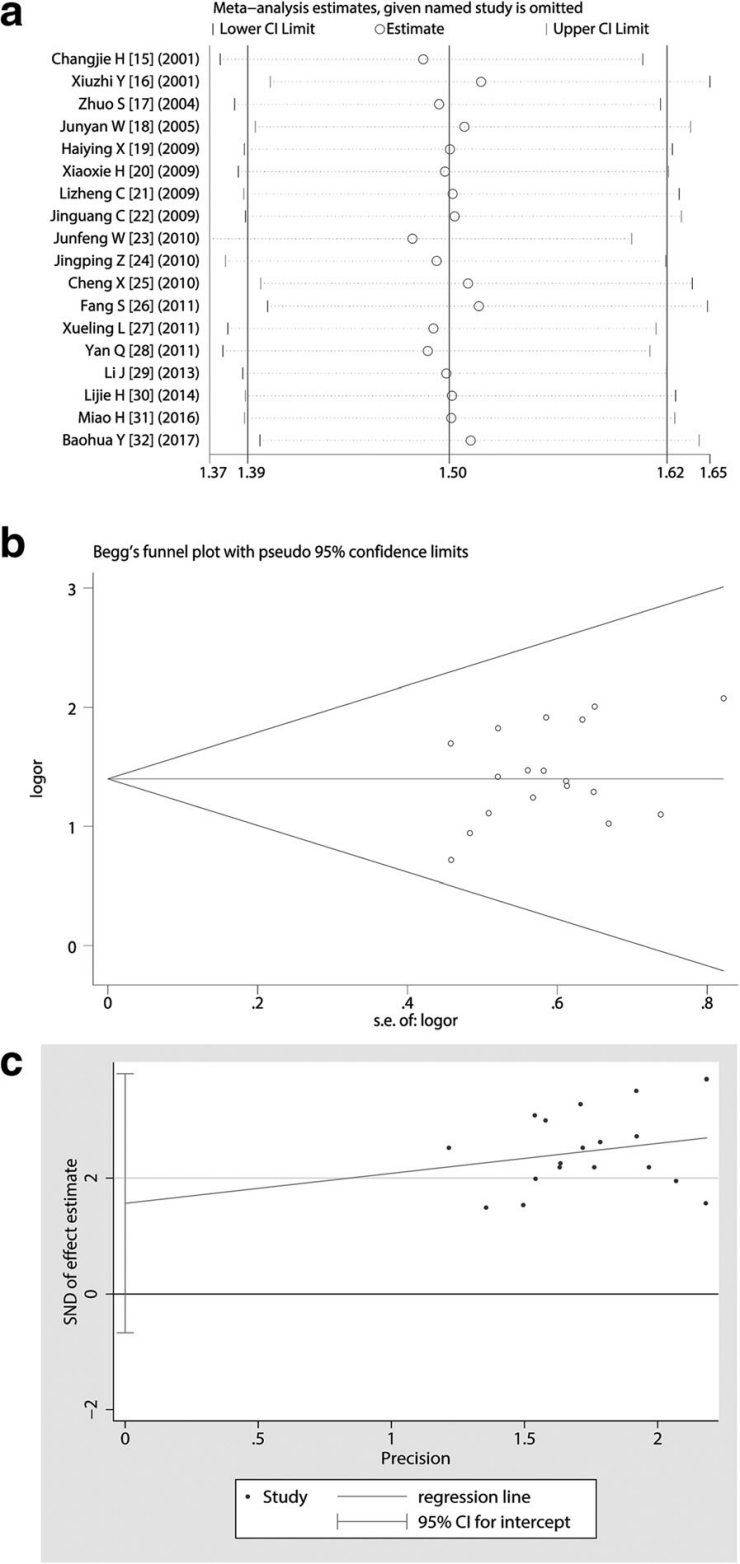
Sensitivity assessment and publication bias analysis. **a** Sensitivity analysis showed that deleting any study did not shake the overall conclusion of meta-analysis; **b** Begg’s test suggested that the included studies did not show a publication bias and the funnel plot seems to be symmetrical; **c** Egger’s test exhibited that *p* value was 0.401, which indicated that the publication bias did not exist

Through a sensitivity analysis, we found that deleting any one study would not shake the overall effect of the meta-analysis. Estimate was distributed from 1.48 to 1.25 (95% CI 1.38 to 1.62). The weight distribution of the study was from 1.92% [22] to 9.92% [25]. Forest maps of sensitivity analysis suggested that the variability of the studies was small and evenly distributed around the 1.50 estimate (Fig. 6a). The above data showed that the stability of this meta-analysis was good, and the conclusions reached on the basis of the above should be more reliable.

### Publication biases in this meta-analysis were less likely to exist

We performed a Begg’s Test and found that the Kendall’s Score (P-Q) was 25, Std. Dev. Of Score was 26.40, z was 0.95 (Pr > | z | = 0.344. Forest plot from Begg’s Test appeared more symmetrical and the included studies were uniformly and symmetrically distributed at the bottom of the funnel plot (Fig. 6b). We also did an Egger’s test and found that t value was 0.86 with 17 degree of free (*p* = 0.401) (95% CI -0.7560045 to 1.792734) (Fig. 6c). Based on the above data, the possibility of publication biases in these studies were very small.

## Discussion

Malignant pleural effusions (MPE) often occur in many cancer patients as the tumor progresses [3]. With the continuous development of molecular medicine, the survival of patients with cancer gradually extended, MPE has become one of the key problem that respiratory physicians, oncologists and thoracic surgeons have to face [33]. Although there are more and more studies on MPE therapy, pleural effusion drainage, intra-thoracic infusion chemotherapy and systemic chemotherapy are the main treatment of MPE [1]. Interleukin-2 (IL-2) is a cytokine with most important functions in the physiology of cell-mediated immunity [12]. IL-2 causes activation, proliferation, and trafficking of T-cells and natural killer cells. When administered locally it may change a non-inflamed tumor into an inflamed tumor, perhaps thereby increasing sensitivity of that tumor to further immune attack [34]. One previous meta-analysis shows that IL-2 or induced killer cells combination therapy displays a considerable efficacy in treating NSCLC and thus improves overall survival. Now IL-2 has been used for the treatment of MPE through thoracic injection. Here, we performed a systemic review and meta-analysis to evaluate the effcacy and safety of IL-2 for patients with MPE through thoracic injection. By retrieving a series of medical databases, based on strict pre-enacted inclusion and exclusion criteria, a total of 18 studies that met the inclusion criteria were recruited in this meta-analysis. We evaluated the study quality of included studies based on the Cochrane Handbook of Systematic Review and the principle of homogeneity in clinical research and found that these studies have good clinical homogeneity. Further heterogeneity analysis also showed that there was no significant heterogeneity among these studies, which indicating that the design and implementation of these studies were well comparable. Thus, we used the fixed effects model of meta-analysis to evaluate efficacy and safety of IL-2 for treating patients with MPE through thoracic injection.

Amazingly, we found that the combination of cisplatin plus IL-2 via thoracic injection significantly increased the ORR (showing an absolute 4.1-fold elevation) as well as DCR (an absolute 7.86-fold increase), as compared with cisplatin alone. This result gives us a message that IL-2 has a role in the treatment of MPE, and when used in combination with cisplatin, it significantly enhances the efficacy to control MPE with synergistic effects. It has been shown that the thoracic injection of IL-2 prolongs the survival of patients with MPE. The possible mechanism is that IL-2 increases the number of CD3 + T cells and NK cells in the pleural space and enhances immunity reaction, reducing the incidence of MPE; the data show that the IL-2 treatment for MPE showed an effective rate of about ~ 59% and no significant side effects [35–37]. A recent study of IL-2 in MPE indicates that IL-2 reduces (PD-1) expression, increases expressions of granzyme B (GzmB) and γ- interferon, and enhances CD8+ T cell proliferation in MPE. In addition, IL-2 reduces carcinoembryonic antigen (CEA) expression in MPE, suggesting that its mechanism of treatment for MPE is closely related to these molecules [35]. Cisplatin is a non-specific cell cycle anti-tumor drug, clinically used for the treatment of MPE. Thoracic injection of cisplatin not only directly kill tumor cells, improve lymphatic circulation, can also lead to chemical pleurisy, pleural adhesions, thereby reducing pleural exudation [38]. We deduce that the combination of cisplatin and IL-2 can play their respective advantages, the two applications can improve the body’s immune function and show a synergistic effect. To confirm this result from another aspect, we compared the no-response rate (NRR) of treatment between cisplatin plus IL-2 versus cisplatin alone through thoracic injection for treating MPE and found that NRR of cisplatin alone was remarkably higher than that of cisplatin plus IL-2, which showed that patients with MPE could get a better benefit from cisplatin plus IL-2 than IL-2 alone.

With the continuous improvement of cancer diagnosis and treatment technology, patients’ survival and cure rates have been significantly improved, so the improvement of QOL of patients has become increasingly important [39]. We compared the QOL between cisplatin plus IL-2 versus cisplatin alone through thoracic injection for treating MPE and noticed that combination perfusion of cisplatin and IL-2 improved the QOL of patients with MPE, as compared with cisplatin alone, which showed an absolute 2.75-fold increase. In recent years, health care has progressively increased its interest in understanding QOL as a crucial and meaningful endpoint, particularly in oncology [40]. Clinically, when cancer patients develop an MPE condition, which means that the tumor has been locally metastasized or disseminated, implying that it is difficult to cure. Patients with MPE mainly manifested as chest pain, dyspnea, weight loss and other cachexia symptoms, however, the effective control of MPE can effectively alleviate the symptoms of patients, reduce pain and improve QOL. Anti-cancer drugs are an important part of many cancer treatments. The research and development of new anti-cancer drugs is one of the hot spots in cancer research. However, some anticancer drugs may also damage healthy cells while damaging cancer cells, leading to side effects [41]. When a drug is used to treat a disease, its side effects are also an important indicator of its performance. The same efficacy of treatment, we are willing to use the method of smaller side effects [42]. In this meta-analysis, we found that despite the combination of IL-2 with cisplatin, the incidence rate on myelotoxicity, nausea/vomiting and chest pain did not show an extra increase, as compared with cisplatin alone. Unfortunately, we found that compared with cisplatin alone, thoracic injection of cisplatin plus IL-2 led to a higher possibility of fever. However, the fever caused by IL-2 was only mild to moderate. After the physical cooling and antipyretics, the symptoms disappeared. IL-2 has been used as a method of immunotherapy for a variety of oncology treatments. Fever is clinically common adverse reaction caused by IL-2, some patients need anti-fever drugs. However, recent tumor immunology studies have shown that elevated temperatures can promote more effective anti-tumor immune responses, which may be more conducive to anti-tumor treatment [43]. Patients with IL-2 are expected to have varying degrees of systemic toxicity. There is evidence that increased doses of IL-2 lead to increased toxicity [44]. Several dosage regiments, including intravenous high doses (720,000 or 600,000 international units/kg), low dose subcutaneous injection, and IL-2 combined with other therapies, have been used for maximum therapeutic benefit. There are currently 60 institutions in North America with high doses of IL-2 for metastatic melanoma and renal cell carcinoma [45]. Currently in China, the recommended dosage of IL-2 for the treatment of MPE is 0.5 to 1 million IU/ m2/ time. The studies included in our meta-analysis were performed in accordance with this dose. Our study showed that this dose indicated a better efficacy and lower side effects. Since these studies did not concern the effects of different doses of IL-2 on side effects, our meta-analysis could not provide a conclusion about how different doses might affect side effects. This also reminds related researchers that this problem should be paid attention to in future research.

Through meticulous sensitivity analysis and publication biases analysis, we found that the studies included in this meta-analysis were of good homogeneity, so the conclusions are stable. However, there are still some shortcomings in these researches. First of all, the dosage of IL-2 was defined 1 to 3 million units in those studies; IL-2 dose showed some differences, suggesting that the IL-2 combined with cisplatin by thoracic injection for MPE treatment needs further standardization. Second, the description of randomized allocation and blinded implementation of included studies is unclear and there may be a risk of selective bias, which may affect the strength of evidence of the findings. Third, because there was no foreign literature to meet the inclusion criteria, there may be a geographical bias. Fourth, to date, there are no multicenter and large sample studies. We hope that in the future there will be more scientifically designed and rigorous RCTs of large samples to provide a reliable basis for the clinical use of IL-2 in treatment of MPE by thoracic injection.

## Conclusions

Due to ethical and drug clinical research restrictions, clinical studies on new drugs are often very cautious. Despite this, this meta-analysis still give us an important message that thoracic injection of cisplatin plus low-dose IL-2 has a better benefit of ORR, DCR and QOL for controlling MPE, compared with cisplatin alone, which means that IL-2 may be one of the options to treat MPE. Especially, except for the fever, the presence of low-dose IL-2 does not have an extra increase on the incidence of other AEs. However, further randomized trials with large population are required to provide more evidence for evaluating the efficacy of IL-2 in the treatment of MPE.

## Abbreviations

AEs: Adverse reactions
CD4 +: Antigen-simulated cluster of differentiation 4
CD8 +: Cluster of differentiation 8
CEA: Carcinoembryonic antigen
CI: Confidence intervals
CNKI: China National Knowledge Infrastructure
CR: Complete response
DCR: Disease control rate
EMBASE: Excerpt Medica Database
GzmB: granzyme B
IL-2: interleukin 2
KPS: karnofsky scores
MPE: Malignant pleural effusion
MPEs: Malignant pleural effusions
NK: Natural killer
NRR: Non-response rate
NSCLC: Non-small cell lung cancer
OR: Odds ratio
ORR: Objective response rate
OS: Overall survival
PD: Progressive disease
PD-1: Programmed cell death 1
PR: Partial response
QOL: Quality of life
RCTs: Randomised controlled trials
RECST: Response evaluation criteria in solid tumors
SD: Stable disease
SFDA: China State Food and Drug Administration
WHO: World Health Organization
